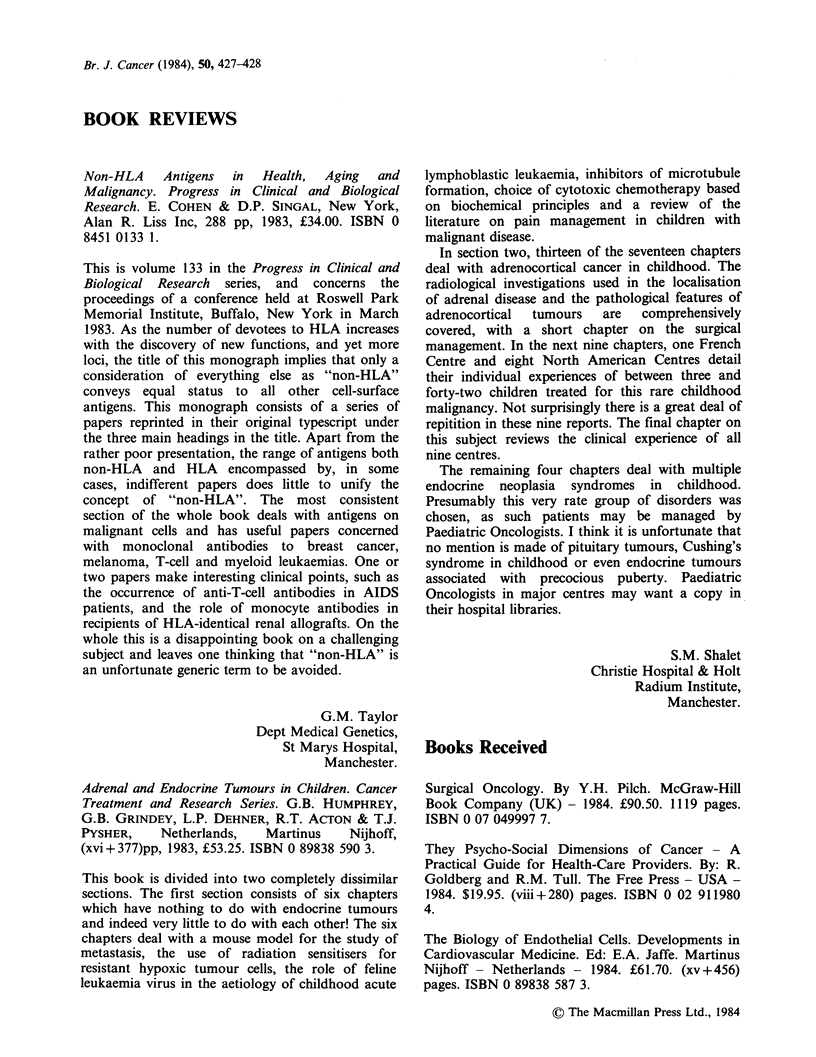# Non-HLA Antigens in Health, Aging and Malignancy. Progress in Clinical and Biological Research

**Published:** 1984-09

**Authors:** G.M. Taylor


*Br. J. Cancer* (1984), **50**, 427–428

### BOOK REVIEWS

*Non-HLA Antigens in Health, Aging and Malignancy. Progress in Clinical and Biological Research.* E. COHEN & D.P. SINGAL, New York, Alan R. Liss Inc, 288 pp, 1983, £34.00. ISBN 0 8451 0133 1.

This is volume 133 in the *Progress in Clinical and Biological Research* series, and concerns the proceedings of a conference held at Roswell Park Memorial Institute, Buffalo, New York in March 1983. As the number of devotees to HLA increases with the discovery of new functions, and yet more loci, the title of this monograph implies that only a consideration of everything else as "non-HLA" conveys equal status to all other cell-surface antigens. This monograph consists of a series of papers reprinted in their original typescript under the three main headings in the title. Apart from the rather poor presentation, the range of antigens both non-HLA and HLA encompassed by, in some cases, indifferent papers does little to unify the concept of "non-HLA". The most consistent section of the whole book deals with antigens on malignant cells and has useful papers concerned with monoclonal antibodies to breast cancer, melanoma, T-cell and myeloid leukaemias. One or two papers make interesting clinical points, such as the occurrence of anti-T-cell antibodies in AIDS patients, and the role of monocyte antibodies in recipients of HLA-identical renal allografts. On the whole this is a disappointing book on a challenging subject and leaves one thinking that "non-HLA" is an unfortunate generic term to be avoided.

G.M. Taylor
Dept Medical Genetics,
St Marys Hospital,
Manchester.